# GotEnzymes2: expanding coverage of enzyme kinetics and thermal properties

**DOI:** 10.1093/nar/gkaf1053

**Published:** 2025-10-31

**Authors:** Bingxue Lyu, Ke Wu, Yuanyuan Huang, Mihail Anton, Xiongwen Li, Sandra Viknander, Danish Anwer, Yunfeng Yang, Diannan Lu, Eduard Kerkhoven, Aleksej Zelezniak, Dan Gao, Yu Chen, Feiran Li

**Affiliations:** Institute of Biopharmaceutical and Health Engineering, Tsinghua Shenzhen International Graduate School, Tsinghua University, Shenzhen 518055,China; Key Laboratory for Industrial Biocatalysis, Ministry of Education, Institute of Biochemical Engineering, Department of Chemical Engineering, Tsinghua University, Beijing 100084,China; Institute of Biopharmaceutical and Health Engineering, Tsinghua Shenzhen International Graduate School, Tsinghua University, Shenzhen 518055,China; Key Laboratory for Industrial Biocatalysis, Ministry of Education, Institute of Biochemical Engineering, Department of Chemical Engineering, Tsinghua University, Beijing 100084,China; State Key Laboratory of Quantitative Synthetic Biology, Shenzhen Institute of Synthetic Biology, Shenzhen Institutes of Advanced Technology, Chinese Academy of Sciences, Shenzhen 518055,China; Department of Life Sciences, Chalmers University of Technology, Gothenburg SE-412 96,Sweden; ELIXIR, Wellcome Genome Campus, Hinxton, Cambridgeshire CB10 1SD,United Kingdom; Institute of Biopharmaceutical and Health Engineering, Tsinghua Shenzhen International Graduate School, Tsinghua University, Shenzhen 518055,China; Key Laboratory for Industrial Biocatalysis, Ministry of Education, Institute of Biochemical Engineering, Department of Chemical Engineering, Tsinghua University, Beijing 100084,China; Department of Life Sciences, Chalmers University of Technology, Gothenburg SE-412 96,Sweden; Department of Life Sciences, Chalmers University of Technology, Gothenburg SE-412 96,Sweden; Institute of Environment and Ecology, Tsinghua Shenzhen International Graduate School, Tsinghua University, Shenzhen 518055,China; Department of Chemical Engineering, Tsinghua University, Beijing 100084,China; Department of Life Sciences, Chalmers University of Technology, Gothenburg SE-412 96,Sweden; Novo Nordisk Foundation Center for Biosustainability, Technical University of Denmark, Lyngby, Kongens 2800,Denmark; SciLifeLab, Chalmers University of Technology, Gothenburg SE-412 96,Sweden; Department of Life Sciences, Chalmers University of Technology, Gothenburg SE-412 96,Sweden; Randall Centre for Cell & Molecular Biophysics, King’s College London, Guy’s Campus, London SE1 1UL,United Kingdom; Institute of Biotechnology, Life Sciences Centre, Vilnius University, Vilnius Sauletekio al. 7 LT10257,Lithuania; Institute of Biopharmaceutical and Health Engineering, Tsinghua Shenzhen International Graduate School, Tsinghua University, Shenzhen 518055,China; State Key Laboratory of Quantitative Synthetic Biology, Shenzhen Institute of Synthetic Biology, Shenzhen Institutes of Advanced Technology, Chinese Academy of Sciences, Shenzhen 518055,China; Institute of Biopharmaceutical and Health Engineering, Tsinghua Shenzhen International Graduate School, Tsinghua University, Shenzhen 518055,China; Key Laboratory for Industrial Biocatalysis, Ministry of Education, Institute of Biochemical Engineering, Department of Chemical Engineering, Tsinghua University, Beijing 100084,China

## Abstract

Enzyme kinetics are fundamental for understanding metabolism, yet experimentally measured parameters remain scarce. To address this gap, we introduce GotEnzymes2, a substantially expanded resource covering 10 765 species, 7.3 million enzymes, and 59.6 million unique entries. Compared with the first version, GotEnzymes2 now integrates both catalytic and thermal parameters, enabling unified predictions of *k*_cat_, *K*_m_,*k*_cat_/*K*_m_, optimal temperature, and melting temperature. This expansion markedly broadens species and enzyme coverage, creating the most comprehensive database of enzyme kinetic and stability parameters to date. To construct the resource, we systematically benchmarked state-of-the-art models for catalytic and thermal parameter prediction, and incorporated the best-performing strategies to ensure accuracy and generalizability. Altogether, GotEnzymes2 provides the community with a powerful resource for data-driven enzyme discovery, design, and engineering, with broad applications in systems biology, metabolic engineering, and synthetic biology. GotEnzymes2 is publicly accessible at https://metabolicatlas.org/gotenzymes.

## Graphical Abstract

**Graphical Abstract:**
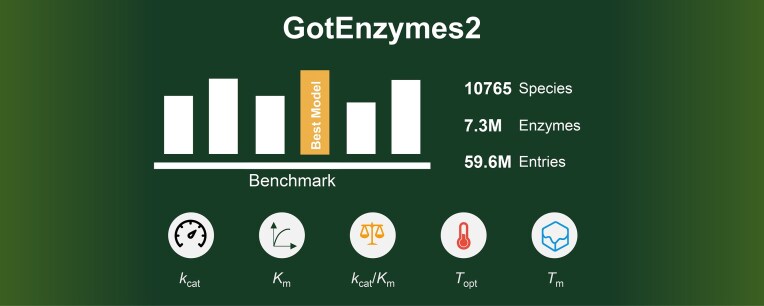

## Introduction

Enzymes, the primary biological catalysts in living organisms, play an essential role in metabolic processes and cellular function [1, 2]. Quantitative characterization of their catalytic efficiency and thermal stability is of both significant theoretical and practical importance for understanding biological metabolism [3, 4], guiding enzyme engineering [5], optimizing industrial bioprocesses, and advancing the field of synthetic biology [6].

Catalytic efficiency is defined by three core kinetic parameters: *k*_cat_ (turnover number), which represents the maximum number of substrate molecules converted by an enzyme active site per unit time; *K*_m_ (Michaelis constant), which represents the substrate concentration required to achieve half of the maximum catalytic rate and measures substrate affinity; and *k*_cat_/*K*_m_ (catalytic efficiency), which estimates overall catalytic performance. In addition, enzymes are characterized by their thermal properties. An enzyme’s optimal temperature (*T*_opt_) defines the temperature at which an enzyme exhibits peak activity. Thermal stability is often characterized by the melting temperature, *T*_m_, which measures the enzyme’s resistance to denaturation at elevated temperatures. Both *T*_opt_ and *T*_m_ are crucial for understanding enzyme function across diverse environments and are particularly important for industrial applications. However, existing databases that record enzyme kinetic parameters and thermal properties, such as BRENDA [7], SABIO-RK [8], and UniProt [9], have limited coverage of kinetic and thermal properties due to scarcity of the experimental data, posing a significant barrier to the *in silico* rational selection and engineering of enzymes for diverse applications [10]. To address this gap, various computational models have been developed in recent years (Table 1). For kinetic parameter prediction, models including DLKcat [11], TurNuP [12], DLTKcat [13], DeepEnzyme [14], Kroll *et al.*’s model (referred to as Boost_KM) [15], UniKP [16], EITLEM-Kinetics [17], and CataPro [18] have been developed. In parallel, models including TOMER [19] and Seq2Topt [20] have been developed for predicting enzyme thermal properties. These diverse methods have significantly advanced the field of enzyme property prediction, yet challenges remain in benchmarking and generalizability across diverse biological contexts.

**Table 1.** Enzyme kinetic and thermal properties prediction model

Model	Parameters	Input	Characteristics
DLKcat [11]	*k*_cat_	Protein sequence and substrate	*k*_cat_ (*R*^2^ = 0.49), integrated in GECKO 3.0
TurNuP [12]	*k*_cat_	Protein sequence and reaction fingerprint	*k*_cat_ (*R*^2^ = 0.44) of an entire reaction, unable to differentiate the *k*_cat_ for each substrate in multi-substrate reactions
DLTKcat [13]	*k*_cat_	Protein sequence, substrate, and temperature	*k*_cat_ at different temperatures (*R*^2^ = 0.66)
DeepEnzyme [14]	*k*_cat_	Protein sequence, substrate, and protein 3D structure	*k*_cat_ (*R*^2^ = 0.58) utilizing protein 3D structure
Boost_KM ^a^ [15]	*K*_m_	Protein sequence and substrate	*K*_m_ (*R*^2^ = 0.53)
UniKP [16]	*k*_cat_, *K*_m_, *k*_cat_/*K*_m_	Protein sequence and substrate	*k*_cat_ (*R*^2^ = 0.67), *K*_m_ (*R*^2^ = 0.60), and *k*_cat_/*K*_m_ (*R*^2^ = 0.56), supports temperature and pH inputs
EITLEM-Kinetics [17]	*k*_cat_, *K*_m_, *k*_cat_/*K*_m_	Protein sequence and substrate	*k*_cat_ (*R*^2^ = 0.72), *K*_m_ (*R*^2^ = 0.69), and *k*_cat_/*K*_m_ (*R*^2^ = 0.68) utilizing transfer learning
CataPro [18]	*k*_cat_, *K*_m_, *k*_cat_/*K*_m_	Protein sequence and substrate	*k*_cat_ (PCC = 0.497), *K*_m_ (PCC = 0.633), and *k*_cat_/*K*_m_ (PCC = 0.413), training on hard set, exhibiting strong robustness
TOMER [19]	*T*_opt_	Protein sequence and optimal growth temperature (OGT)	*T*_opt_ (*R*^2^ = 0.632)
Seq2Topt [20]	*T*_opt_, *T*_m_	Protein sequence	*T*_opt_ (*R*^2^ = 0.57) and *T*_m_ (*R*^2^ = 0.64)

a Here, we use Boost_KM to refer to the model developed by Kroll et al.

Benchmarking enzyme prediction models is difficult due to inconsistent datasets, heterogeneous evaluation metrics, and the variable ability of models to generalize across biologically relevant conditions. Existing approaches often lack rigorous assessment of performance on low-homology sequences and in predicting mutational effects, which are two critical aspects for enabling broader applicability. The absence of standardized evaluations across these aspects has hindered both methodological refinement and real-world deployment. To address this, we propose a three-step strategy: first, we retrain existing models on all kinetic parameters (*k*_cat_, *K*_m_, *k*_cat_/*K*_m_) and thermal properties (*T*_opt_, *T*_m_) using a unified dataset to assess the accuracy, generalizability, and mutational prediction capability, respectively; second, we combine diverse feature representations (e.g. pretrained protein language models) with machine or deep learning model architectures to optimize prediction performance; third, we apply the best-performing models to systematically update and expand the GotEnzymes database [21] with large-scale predictions of kinetic and thermal properties across a diverse set of enzymes and organisms, thereby creating a comprehensive resource for enzyme research and engineering.

## Materials and methods

### Dataset acquisition

The EITLEM-Kinetics dataset contains kinetic data for multiple enzyme-substrate reactions, including 34 429 *k*_cat_, 28 664 *K*_m_, and 13 388 *k*_cat_/*K*_m_. These data provide important support for reproducing the DLKcat, UniKP (*k*_cat_, *K*_m_, *k*_cat_/*K*_m_), and Boost_KM models. During data processing, for reactions as inputs in TurNuP, we used EC numbers annotated in the EITLEM-Kinetics datasets to fill in the reaction completeness, ensuring data accuracy and consistency. For DLTKcat, which requires temperature information, we referenced the temperature data included in the BRENDA [7] and SABIO-RK [8] databases to fill in the necessary temperature parameters. For entries lacking thermal parameters in BRENDA and SABIO-RK, we excluded them from the dataset. For protein structure information, we predicted the 3D structures of all protein sequences using ESMFold [22]. The *T*_opt_ dataset (*n* = 2917) was obtained from the GitHub repository of TOMER, which was originally obtained from the BRENDA database. To address the *T*_opt_ imbalance, we doubled the entries with high *T*_opt_ (≥80°C) by randomly duplicating existing points in this range. This creates a more balanced dataset, reducing bias toward lower *T*_opt_ values and improving predictions for high-temperature enzymes [19, 20]. The training and test datasets of thermal stability (*T*_m_) were obtained from DeepTM [23] and Meltome Atlas [24]. The *T*_m_ training and test datasets had 25 399 and 6350 entries, respectively.

### Calculation of protein identity and substrate similarity

We used the MMseqs2 [25] to calculate the identity of protein sequences and the *FingerprintSimilarity* function from *RDKit* to calculate the similarity between substrates.

## Results

### Comparison of different enzyme kinetic and thermal property prediction models on unified datasets

**Figure 1.**
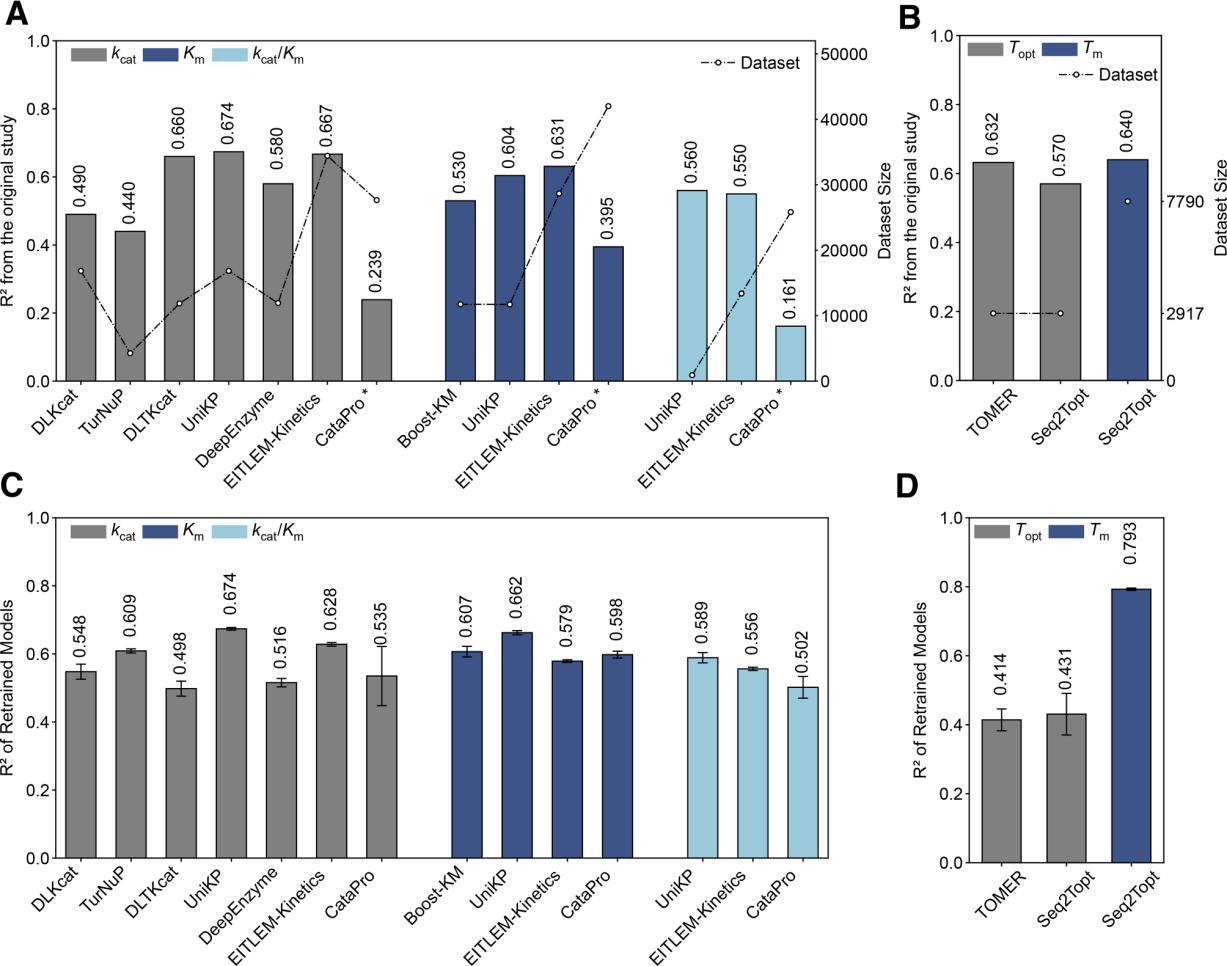
Performance of retrained enzyme kinetic parameter and thermal properties prediction models on unified datasets. **(A)** The dataset size of different enzyme kinetics prediction models and their reported *R*^2^ values. It should be noted that the *R*^2^ for CataPro was calculated using predictions on the test dataset and the corresponding labels. **(B)** The dataset size of different enzyme thermal property prediction models and their reported *R*^2^ values. **(C)***R*^2^ of different retrained kinetic parameter prediction models on the EITLEM-Kinetics datasets. **(D)***R*^2^ of different retrained thermal properties prediction models on the *T*_opt_ and *T*_m_ datasets. Error bars represent the standard deviation of the test performance over five random train-test splits of the dataset (*n* = 5). * To be noted here, CataPro employs an unbiased dataset and splits the training and test sets under protein sequence similarity control. This more challenging strategy results in lower *R*^2^ values compared to random splitting of other models.

We began by collecting kinetic parameter prediction models with available code for both enzyme kinetic parameters (*k*_cat_, *K*_m_, *k*_cat_/*K*_m_) and thermal properties (*T*_opt_, *T*_m_), which exhibited significant differences in their original datasets and reported performance (Fig. 1A and B). To benchmark the performance of kinetic prediction models, we adopted the EITLEM-Kinetics datasets to retrain them, since it is currently the largest in scale, integrating relevant data from UniProt [9], BRENDA [7], and SABIO-RK [8]. This dataset contains 34 429 enzyme–substrate pairs for *k*_cat_, 28 664 enzyme–substrate pairs for *K*_m_, and 13 388 enzyme–substrate pairs for *k*_cat_/*K*_m_ (Fig. 1A). In all three datasets, mutants account for ~40% of all entries (Supplementary Fig. S1A), enabling evaluation of model sensitivity to sequence perturbations. These datasets cover 8000 protein types and 3000 substrates (Supplementary Fig. S1B and C). The *k*_cat_, *K*_m_, and *k*_cat_/*K*_m_ values follow a log-normal distribution (Supplementary Fig. S1D). To evaluate *T*_opt_ and *T*_m_ prediction models, we used the datasets from TOMER [19], DeepTM [23], and Meltome Atlas [24], which contain 2917 *T*_opt_ entries and 31 749 *T*_m_ entries (Supplementary Fig. S1E).

Most existing kinetic models are trained using protein sequence and substrate inputs, which allows for direct retraining with the EITLEM-Kinetics datasets. However, models such as DLTKcat require temperature information, while DeepEnzyme depends on structural information. To accommodate these requirements, we collected the corresponding temperature data through databases (i.e. UniProt [9], BRENDA [7]) and structural data from protein structure prediction models (i.e. ESMFold [22]). In addition, TurNuP was trained using protein sequences and reaction fingerprints, requiring us to extend the dataset with reaction data from BRENDA [7]. Similarly, for *T*_opt_ and *T*_m_ models, we retrained models only when the original training code was available and inputs were limited to either protein sequence alone or in combination with OGT.

After retraining, UniKP (*k*_cat_) and EITLEM-Kinetics (*k*_cat_) performed the best for *k*_cat_ prediction, achieving Coefficient of Determination (*R*^2^) values of 0.674 and 0.628, respectively (Fig. 1C). For *K*_m_ prediction, the retrained Boost_KM, UniKP, EITLEM-Kinetics, and CataPro achieved *R*^2^ values of 0.607, 0.662, 0.579, and 0.598, respectively (Fig. 1C). For *k*_cat_/*K*_m_ prediction, the retrained UniKP (*k*_cat_/*K*_m_) outperformed EITLEM-Kinetics (*k*_cat_/*K*_m_) and CataPro (*k*_cat_/*K*_m_), with *R*^2^ values of 0.589, 0.556, and 0.502, respectively (Fig. 1C). The overall better performance of the *k*_cat_ prediction compared to *K*_m_ and *k*_cat_/*K*_m_ may be attributed to its larger dataset size compared to those for *K*_m_ and *k*_cat_/*K*_m_. Additionally, the *R*^2^ values of Boost_KM and TurNuP showed improvement compared to their original reports, increasing by 0.08 and 0.17 (compared to original report), respectively, further showing the positive impact of dataset expansion on model accuracy.

For *T*_opt_ prediction, TOMER [19] and Seq2Topt [20] were chosen due to the code availability for retraining. TOMER is a machine learning model that takes both sequence and OGT as input features, while Seq2Topt is a deep learning model that uses only sequences as input (Fig. 1D). For *T*_m_ prediction, only Seq2Topt was retrained (Fig. 1D), and its results outperformed the originally reported performance in its publication. The performance of the retrained models was evaluated using the *R*^2^, Pearson’s Correlation Coefficient (PCC), Mean Absolute Error, Spearman Correlation, and Root Mean Square Error, as shown in Supplementary Table S1.

### Evaluation of the generalization ability of enzyme kinetics parameters and thermal property prediction models

**Figure 2.**
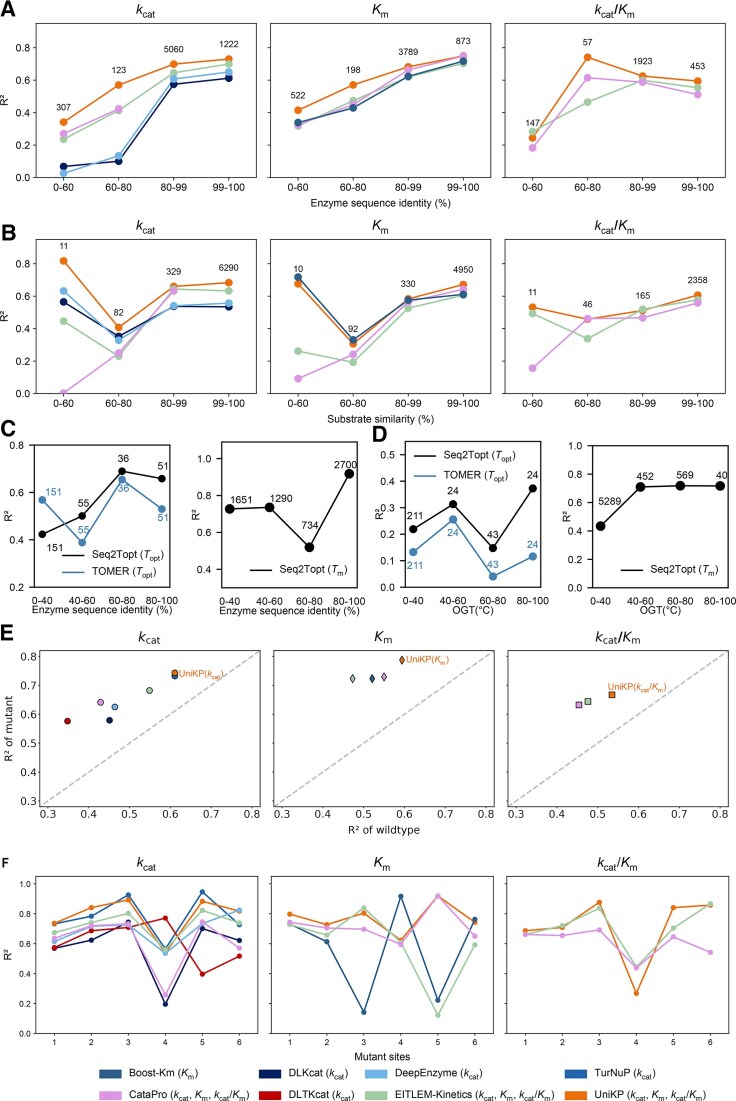
Generalization capabilities of the retrained enzyme kinetic parameter and thermal properties prediction models in the dimensions of protein identity and substrate similarity and performance of the enzyme kinetic parameter models in predicting mutants. Generalization ability of the retrained *k*_cat_, *K*_m_, and *k*_cat_/*K*_m_ prediction models evaluated across **(A)** enzyme sequence identity and **(B)** substrate similarity. **(C)** Generalization ability of the retrained *T*_opt_ and *T*_m_ prediction model. **(D)** Performance of the retrained *T*_opt_ and *T*_m_ prediction model in different OGT intervals. **(E)***R*^2^ of the retrained model predictions for wild-type and mutants on the test set. Here, circles represent the *k*_cat_ model, diamonds represent the *K*_m_ model, and squares represent *k*_cat_/*K*_m_. **(F)***R*^2^ of the retrained model predictions for mutants with varying numbers of mutation sites on the test set.

We systematically evaluated the generalization ability of models for predicting enzyme kinetics and thermal properties, uniquely assessing performance across both protein sequence identity and substrate similarity. For kinetics models, as can be expected, performance declined with decreasing similarity on both axes, with retrained UniKP and Boost_KM showing the most robust generalization for *k*_cat_/*K*_m_ and *K*_m_ predictions, respectively (Fig. 2A and B and Supplementary Fig. S2). We therefore propose that this dual-axis evaluation should become a standard for assessing generalization. In contrast, models predicting thermal properties (*T*_opt_ and *T*_m_) demonstrated stable performance across a wide range of sequence identities, indicating strong generalization even to distant proteins (Fig. 2C) and in different OGT ranges (Fig. 2D).

### Evaluation of enzyme kinetic parameter prediction models on mutants

To assess the models’ utility for enzyme engineering, we evaluated their performance on predicting the kinetic parameters of mutants. The retrained UniKP model was superior, achieving high *R*^2^ values on the mutant dataset for *k*_cat_ (*R*^2^ = 0.743), *K*_m_ (*R*^2^ = 0.787), and *k*_cat_/*K*_m_ (*R*^2^ = 0.667) (Fig. 2E). This high performance was maintained even as the number of mutation sites increased (Fig. 2F). Critically, leading models could also accurately predict the directional impact of mutations on activity; for instance, UniKP (*k*_cat_) predicted whether a mutation would increase or decrease *k*_cat_ with 87.3% accuracy. These findings validate the models’ robustness for variant prediction and highlight their potential to guide rational enzyme design. Further details on comparative performance and directional accuracy are available in the supplementary materials (Supplementary Fig. S3).

### Optimal module combinations for enzyme kinetic and thermal parameter prediction

**Figure 3.**
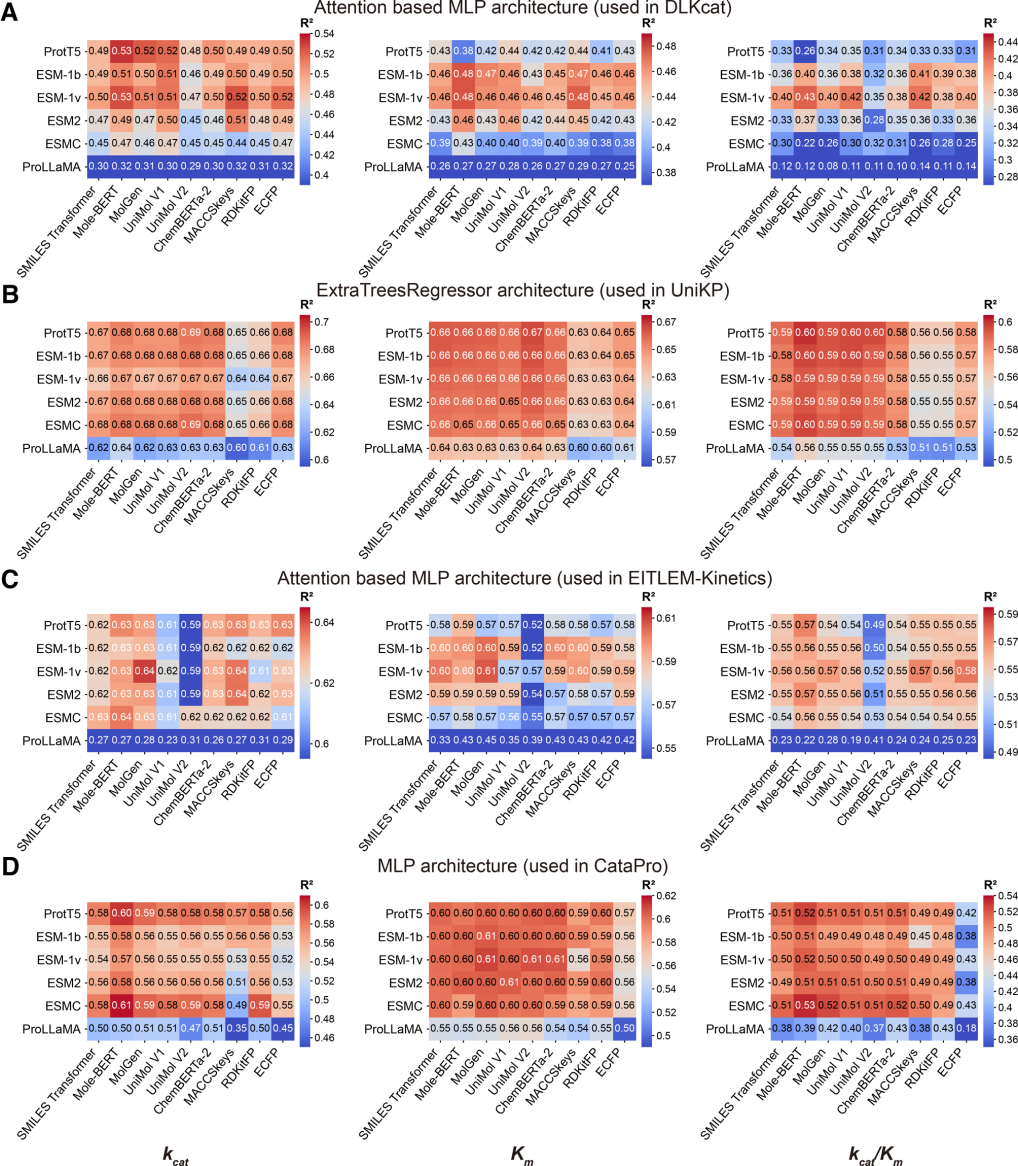
Performance comparison of 216 model configurations. Heatmap showing the *R*^2^ values on the test set for *k*_cat_, *K*_m_, and *k*_cat_/*K*_m_ prediction across all combinations of protein representations, substrate representations, and model architectures. **(A)** Attention-based MLP architecture (used in DLKcat). **(B)** ExtraTreesRegressor architecture (used in UniKP). **(C)** Attention-based MLP architecture (used in EITLEM-Kinetics). **(D)** MLP architecture (used in CataPro).

**Figure 4.**
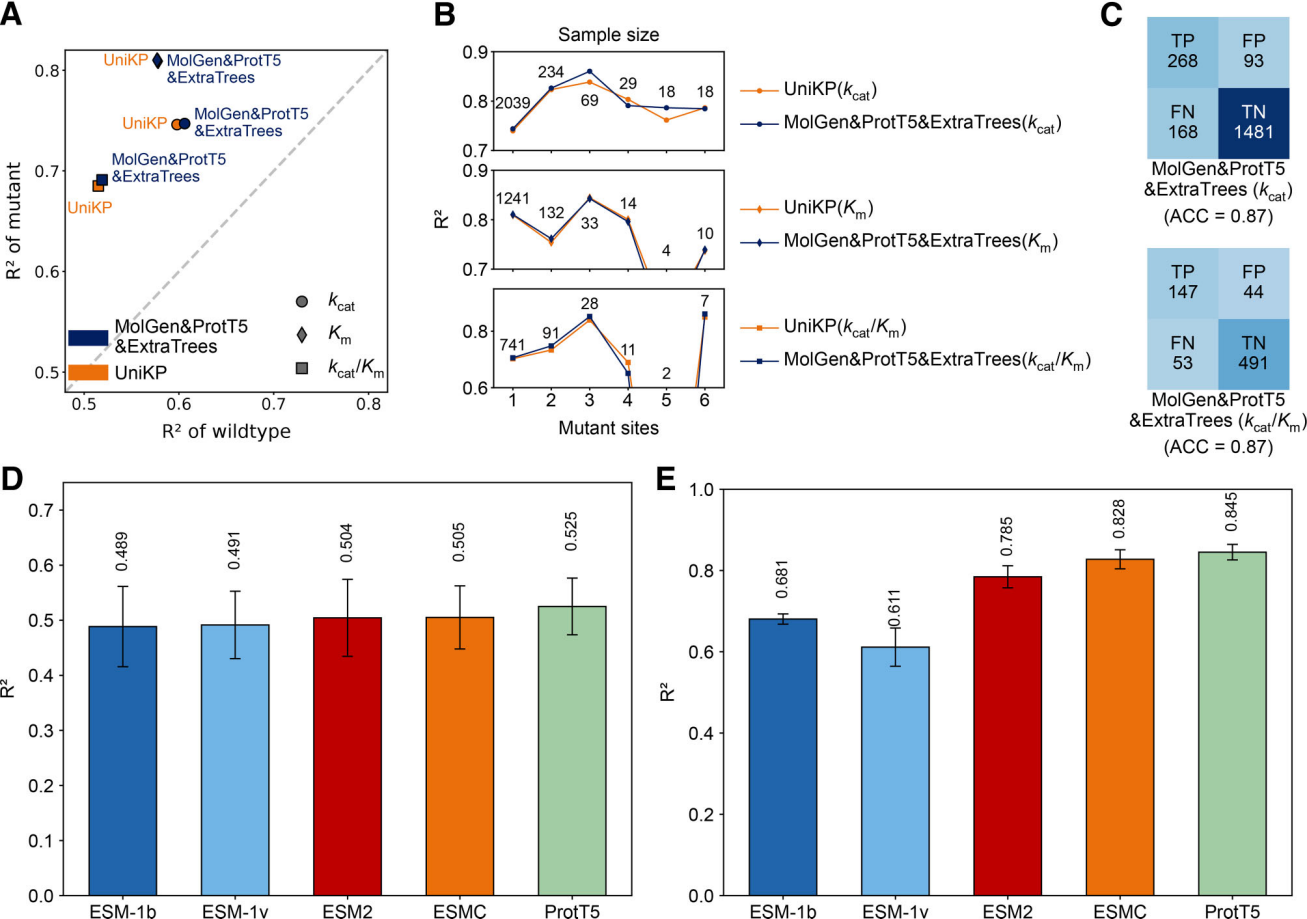
Performance of the optimal combined model. **(A)** Comparison of the *R*^2^ for mutation predictions between the optimal combined model and retrained UniKP (*k*_cat_, *K*_m_, *k*_cat_/*K*_m_) on the test set. Here, circles represent the *k*_cat_ model, diamonds represent the *K*_m_ model, and squares represent *k*_cat_/*K*_m_. **(B)** Comparison of mutation prediction performance across different numbers of mutation sites between the optimal combined model and retrained UniKP (*k*_cat_, *K*_m_, *k*_cat_/*K*_m_) on the test set. **(C)** Comparison of mutation direction prediction performance between the optimal combined model and retrained UniKP (*k*_cat_, *K*_m_, *k*_cat_/*K*_m_). **(D)** Performance of combinations of *T*_opt_ model. **(E)** Performance of *T*_m_ model combinations. Error bars represent the standard deviation of the test performance over five random train-test splits of the dataset (*n* = 5).

To identify the most effective predictive models, we performed a systematic combinatorial screen of key modules, including protein representations, substrate representations, and model architectures (Table 2). For enzyme kinetics, an extensive benchmark of 216 unique configurations revealed that a machine learning architecture (ExtraTrees) paired with large language model representations (ProtT5 for proteins, MolGen for substrates) surpassed existing deep learning models at the current data scale (Fig. 3A–D). This optimal combination, ProtT5&MolGen&ExtraTrees, demonstrated superior performance over all retrained published models, particularly in predicting the parameters of mutants (Fig. 4A–C). Applying a similar strategy to enzyme thermal properties, we identified the combination of ProtT5 and the Seq2Topt architecture as the top performer, which improved *R*^2^ by 0.09 for *T*_opt_ (compared to retrained result) and 0.20 for *T*_m_ over previous state-of-the-art models (Fig. 4D and E).

**Table 2.** Common protein and substrate representations and model architectures

	Substrate representation	Protein representation	Model architecture
Approach	RDKitFP		
	ECFP		
	MACCSkeys FP	ESM-1b[26]	UniKP (ExtraTreesRegressor)
	Mole-BERT [27]	ESM-1v[28]	DLKcat [attention based multilayer perceptron (MLP)]
	ChemBERTa-2 [29]	ESM2 [22]	EITLEM-Kinetics (attention based MLP)
	UniMol V1 [30]	ESM C^a^	CataPro (MLP)
	UniMol V2 [31]	ProtT5 [32]	
	MolGen [33]	ProLLaMA [34]	
	SMILES Transformer [35]		

a ESM C was from https://www.evolutionaryscale.ai/blog/esm-cambrian

### Expansion of the GotEnzymes database

**Figure 5.**
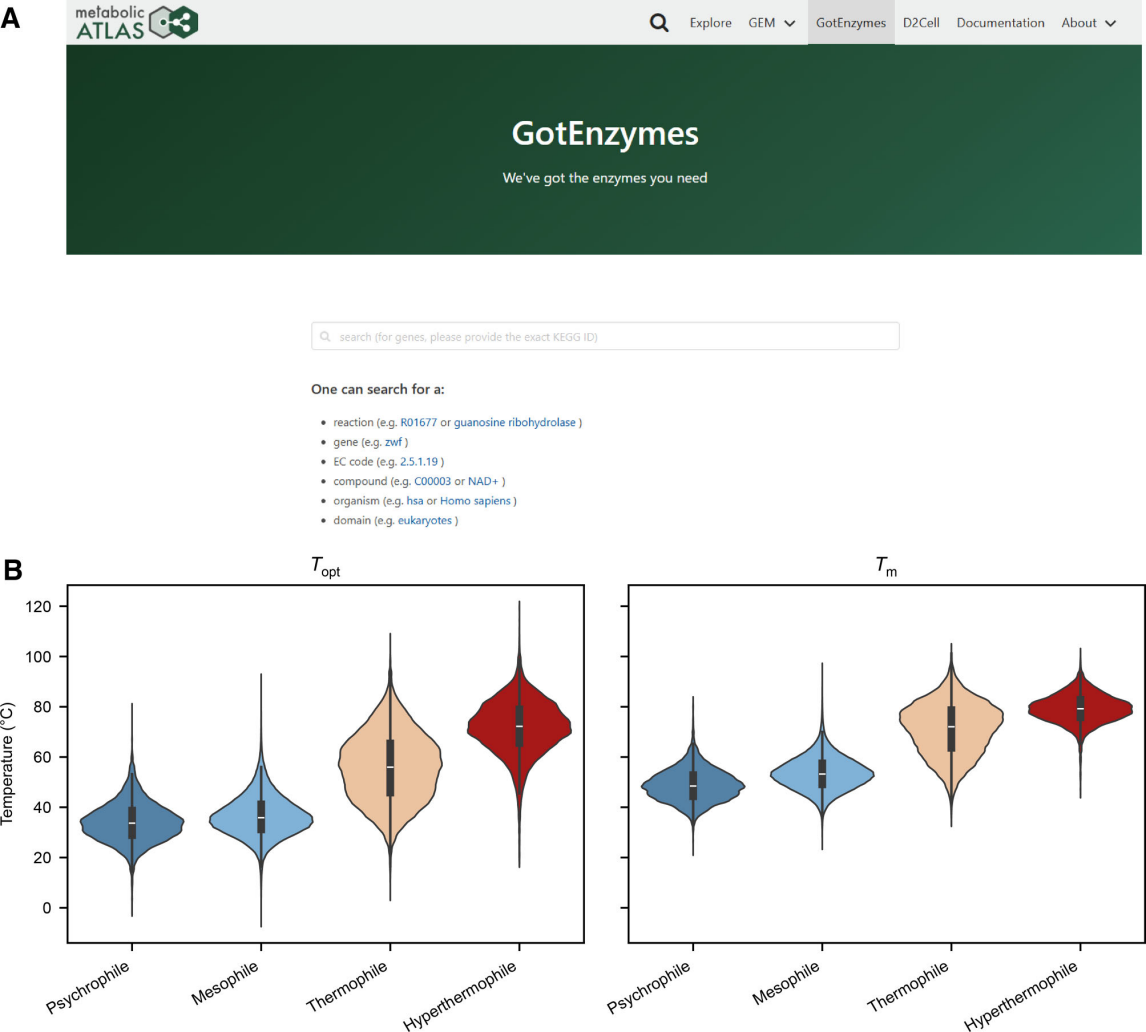
Overview of the GotEnzymes2 database. **(A)** User interface of GotEnzymes2. **(B)** Global analysis of enzyme thermal properties. The sample sizes of organisms were *n* = 19 for psychrophiles, *n* = 5696 for mesophiles, *n* = 253 for thermophiles, and *n* = 61 for hyperthermophiles. The inner box represents the interquartile range (from lower to upper quartile). The central line is the median, and whiskers extend to 1.5× the interquartile range.

The original GotEnzymes database encompassed predicted *k*_cat_ values for 25 million enzyme–substrate pairs, covering 5.8 million enzymes from 8099 species. To further expand the dataset, we updated the species list based on the latest KEGG [36] database, increasing the total number of species to 10 765, the number of enzymes to 7.3 million, and the number of enzyme–substrate pairs to 59.6 million in GotEnzymes2 (Table 3). Additionally, we substantially enriched the range of annotated properties. Using our optimal combined enzyme kinetic model (ProtT5&MolGen&ExtraTrees), we extended predictions to include *k*_cat_, *K*_m_, and *k*_cat_/*K*_m_ parameters. For enzyme thermal properties, we employed the best-performing model (ProtT5&Seq2Topt) to predict *T*_opt_ and *T*_m_ (Fig. 5A). These updates transform GotEnzymes2 into a comprehensive and multi-parameter enzyme property resource, facilitating downstream applications in metabolic engineering, enzyme design, and synthetic biology.

**Table 3.** Comparison between GotEnzymes and GotEnzymes2

	GotEnzymes	GotEnzymes2
Species	8099	10 765
Enzymes (million)	5.8	7.3
Entries (million)	25	59.6
Parameters	*k*_cat_	*k*_cat_, *K*_m_, *k*_cat_/*K*_m_, *T*_opt_, and *T*_m_

### Global analysis of enzyme thermal properties

For our global analysis of enzyme thermal properties, to classify the enzymes into thermal categories, we used the OGT of their respective source organisms. This OGT information was sourced from the GOSHA database [37] and linked to our dataset via organism name mapping between GOSHA and KEGG. The sample sizes of organisms were *n* = 19 for psychrophiles, *n* = 5696 for mesophiles, *n* = 253 for thermophiles, and *n* = 61 for hyperthermophiles. As shown in Fig. 5B, the distributions of optimal reaction temperature (*T*_opt_) and melting temperature (*T*_m_) for these enzyme groups are clearly distinct. Enzymes from psychrophiles and mesophiles, which are adapted to colder environments, exhibit lower thermal characteristics. Specifically, psychrophilic enzymes display the lowest temperature profiles, while mesophilic enzymes typically have *T*_opt_ values clustered in the 30°C–50°C range with correspondingly moderate *T*_m_ values. While enzymes from thermophilic and hyperthermophilic organisms possess significantly higher *T*_opt_ and *T*_m_ values. Their *T*_opt_ values are generally above 70°C, with some hyperthermophilic enzymes showing peak activity near 100°C, and their elevated *T*_m_ values reflect their enhanced thermal stability.

### Case study: data-driven sourcing of a thermostable biocatalyst

The industrial modification of starch requires highly thermostable glycogen branching enzymes (GBE, EC 2.4.1.18), as many existing candidates exhibit insufficient stability at high temperatures. The GotEnzymes2 database is designed to address this challenge directly.

Instead of performing laborious literature searches, a user can simply query for EC number “2.4.1.18” within the database and sort the results by melting temperature (*T*_m_) in descending order. This process rapidly generates a shortlist of top-ranking, hyper-thermostable enzymes, providing ideal starting points for protein engineering. This data-driven workflow can significantly accelerate a project’s initial phase. For instance, the GBE with UniProt ID O50094 (top 0.2%) could be efficiently identified through this method and selected for subsequent directed mutagenesis [38].

## Discussion

Recent years have witnessed substantial progress in the prediction of enzyme properties, including kinetic parameters (*k*_cat_, *K*_m_, *k*_cat_/*K*_m_) and thermal properties (*T*_opt_, *T*_m_), which are crucial for enzyme-constrained modeling and engineering. However, differences in datasets and model performance hinder reproducibility, benchmarking, and widespread adoption. Here, we addressed these limitations through a comprehensive benchmarking framework by retraining leading models on unified large-scale datasets. For kinetic predictions, retrained versions of UniKP and EITLEM-Kinetics emerged as top performers. For thermal properties, Seq2Topt outperformed others after retraining. To assess real-world applicability, we evaluated model generalization to divergent sequences and substrates, as well as performance on mutant enzymes. Notably, retrained UniKP exhibited strong generalization and maintained high accuracy across both wild-type and mutant datasets. Importantly, UniKP, EITLEM-Kinetics, and DeepEnzyme accurately predicted mutation effects, a critical feature for enzyme design. Thermal models showed stable performance across low-homology sequences, suggesting an ability to capture more global determinants of thermostability. To optimize further, we combined advanced protein and molecular representations (e.g. ProtT5, MolGen) with different model architectures. The ProtT5&MolGen&ExtraTrees model improved kinetic predictions, especially for mutants, while ProtT5&Seq2Topt enhanced *T*_opt_ and *T*_m_ prediction. These advances enabled a major update to GotEnzymes, expanding species coverage from 8099 to 10 765 and enzyme–substrate pairs from 25 million to 59.6 million, now including *k*_cat_, *K*_m_, *k*_cat_/*K*_m_, *T*_opt_, and *T*_m_.

In conclusion, our study presents a unified benchmarking framework for enzyme property prediction, identifies optimal model configurations through extensive modular evaluation, and delivers a significantly expanded GotEnzymes2 database encompassing high-accuracy predictions for catalytic and thermal parameters across a broad phylogenetic landscape. However, several challenges remain despite significant advances. Model performance remains constrained by the quality of available data and the limited integration of structural information. Additionally, model outputs can vary substantially across architectures, posing a challenge for interpretability and reliability. Future efforts should prioritize the curation of higher-quality datasets, inclusion of underrepresented enzyme classes, and incorporation of structure-aware representations to drive more consistent and mechanistically grounded predictions. Ultimately, the continued expansion of publicly available, experimentally verified enzyme kinetic and thermal stability data will be the most crucial element for training next-generation models with even higher accuracy and broader applicability.

## Supplementary Material

gkaf1053_Supplemental_File

## Data Availability

The unified dataset used for retraining the kinetic parameter prediction models is available via EITLEM-Kinetics (https://github.com/XvesS/EITLEM-Kinetics). The dataset used for retraining the thermal properties prediction models is obtained from DeepTM (https://github.com/liimy1/DeepTM), TOMER (https://github.com/jafetgado/tomer/), and Meltome Atlas (https://meltomeatlas.proteomics.wzw.tum.de/master_meltomeatlasapp/). The KEGG database (https://www.genome.jp/kegg/) was used for the GotEnzymes2 database (https://digitallifethu.com/gotenzymes). The authors declare that all data supporting the findings and enabling the reproduction of all figures in this study are available within the paper and its Supplementary Information. Source data are provided with this paper. All data used in this study can be accessed at https://github.com/LiLabTsinghua/GotEnzymes2. To facilitate further use, we have made all the codes and detailed instructions available in our GitHub repository, located at https://github.com/LiLabTsinghua/GotEnzymes2.
