# Supplementary material for: GotEnzymes2: expanding coverage of enzyme kinetics and thermal properties
Source: Nucleic Acids Res. 2025 Oct 31;54(D1):D583–92. doi: 10.1093/nar/gkaf1053 (PMC12807647; doi:10.1093/nar/gkaf1053)
Supplement: gkaf1053_Supplemental_File [file gkaf1053_supplemental_file.docx]

**Supplementary Information**

Bingxue Lyu^1, 2, #^, Ke Wu^1, 2, #^, Yuanyuan Huang^3^, Mihail Anton^4, 5^, Xiongwen Li^1, 2^, Sandra Viknander^4^, Danish Anwer^4^, Yunfeng Yang^6^, Diannan Lu^7^, Eduard Kerkhoven^4, 8, 9^, Aleksej Zelezniak^4, 10, 11^, Dan Gao^1,^ *, Yu Chen^3,^ *, Feiran Li^1, 2,^ *

1 Institute of Biopharmaceutical and Health Engineering, Tsinghua Shenzhen International Graduate School, Tsinghua University, Shenzhen 518055, China

2 Key Laboratory for Industrial Biocatalysis, Ministry of Education, Institute of Biochemical Engineering, Department of Chemical Engineering, Tsinghua University, Beijing 100084, China

3 State Key Laboratory of Quantitative Synthetic Biology, Shenzhen Institute of Synthetic Biology, Shenzhen Institutes of Advanced Technology, Chinese Academy of Sciences, Shenzhen 518055, China

4 Department of Life Sciences, Chalmers University of Technology, Gothenburg SE-412 96, Sweden

5 ELIXIR, Wellcome Genome Campus, Hinxton, Cambridgeshire CB10 1SD, United Kingdom

6 Institute of Environment and Ecology, Tsinghua Shenzhen International Graduate School, Tsinghua University, Shenzhen 518055, China

7 Department of Chemical Engineering, Tsinghua University, Beijing 100084, China

8 Novo Nordisk Foundation Center for Biosustainability, Technical University of Denmark, Lyngby 2800 Kongens, Denmark
9 SciLifeLab, Chalmers University of Technology, Gothenburg SE-412 96, Sweden

10 Randall Centre for Cell & Molecular Biophysics, King’s College London, Guy’s Campus, London, UK

11 Institute of Biotechnology, Life Sciences Centre, Vilnius University, Vilnius, Lithuania

# These authors contributed equally to this work: Bingxue Lyu, Ke Wu.

* Corresponding author:

Feiran Li, Email: [feiranli@sz.tsinghua.edu.cn](mailto:feiranli@sz.tsinghua.edu.cn)

Yu Chen, Email: [y.chen3@siat.ac.cn](mailto:y.chen3@siat.ac.cn)

Dan Gao, Email: [gao.dan@sz.tsinghua.edu.cn](mailto:gao.dan@sz.tsinghua.edu.cn)

**
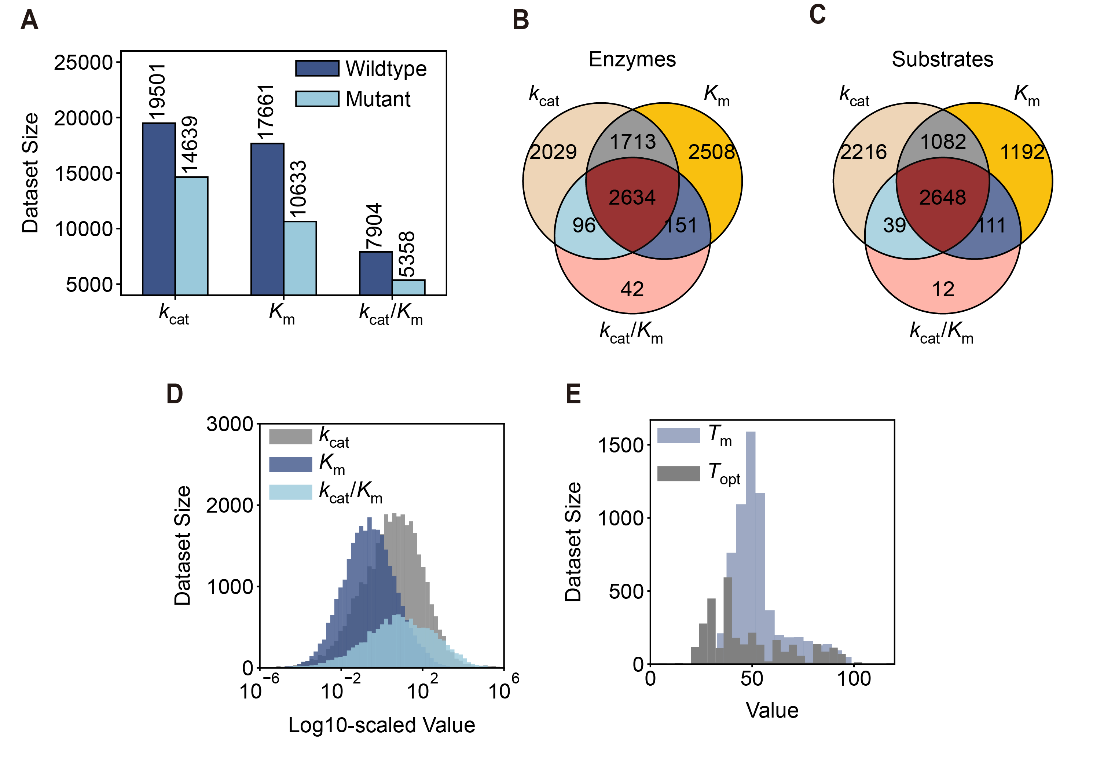
**

**Supplementary Fig. S1 Analyses of datasets.** (A) Composition of the EITLEM-Kinetics datasets. Counts of (B) enzymes and (C) substrates in the EITLEM-Kinetics datasets. (D) Distribution of *k*_cat_, *K*_m_, and *k*_cat_/*K*_m_ values in the EITLEM-Kinetics datasets. (E) Distribution of *T*_opt_ and *T*_m_ values in the TOMER and DeepTM datasets.

**
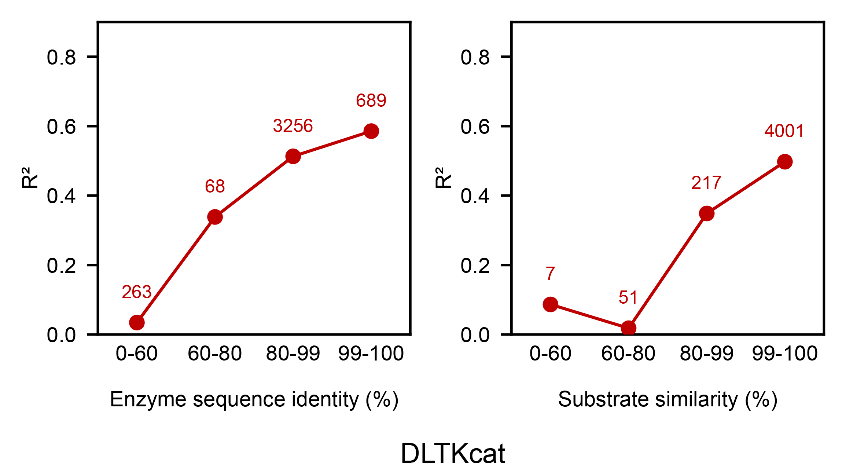
**

**Supplementary Fig. S2 Generalization capabilities of DLTKcat.**


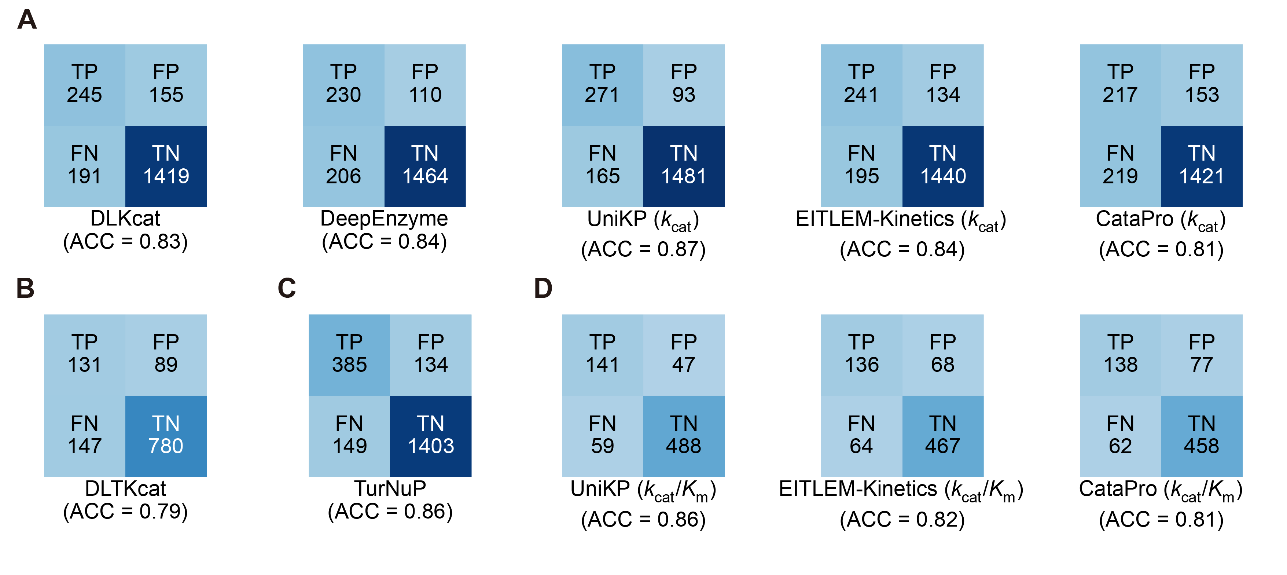


**Supplementary Fig. S3 Mutation direction prediction of *k*_cat_ models (A)-(C) and *k*_cat_/*K*_m_ models (D).**

**Supplementary Table S1 Performance of retrained enzyme parameter prediction models**

| **Parameter** | **Method** | **R^2^ reported in original publication** | **Retrained Model Performance** | | | | |
| --- | --- | --- | --- | --- | --- | --- | --- |
|  |  |  | **R^2^** | **PCC** | **MAE** | **RMSE** | **SCC** |
| ***k*_cat_** | DLKcat | 0.49 | 0.548 | 0.729 | 0.734 | 1.057 | 0.729 |
|  | TurNuP | 0.44 | 0.609 | 0.791 | 0.657 | 0.944 | 0.818 |
|  | DLTKcat | 0.66 | 0.498 | 0.705 | 0.778 | 1.092 | 0.682 |
|  | UniKP (*k*_cat_) | 0.67 | **0.674** | **0.821** | 0.590 | **0.871** | **0.821** |
|  | DeepEnzyme | 0.58 | 0.516 | 0.743 | 0.706 | 1.029 | 0.742 |
|  | EITLEM-Kinetics (*k*_cat_) | 0.66 | 0.628 | 0.804 | **0.582** | 0.927 | 0.792 |
|  | CataPro (*k*_cat_) | 0.24 | 0.535 | 0.760 | 0.708 | 1.010 | 0.761 |
| ***K*_m_** | UniKP (*K*_m_) | 0.60 | **0.662** | **0.813** | **0.538** | **0.746** | **0.811** |
|  | Boost_KM | 0.53 | 0.607 | 0.791 | 0.657 | 0.806 | 0.771 |
|  | EITLEM-Kinetics (*K*_m_) | 0.63 | 0.579 | 0.804 | 0.582 | 0.823 | 0.771 |
|  | CataPro (*K*_m_) | 0.40 | 0.598 | 0.783 | 0.588 | 0.808 | 0.790 |
| ***k*_cat_/*K*_m_** | UniKP (*k*_cat_/*K*_m_) | 0.56 | **0.589** | **0.774** | 0.796 | **1.110** | **0.766** |
|  | EITLEM-Kinetics (*k*_cat_/*K*_m_) | 0.55 | 0.556 | 0.754 | **0.787** | 1.132 | 0.741 |
|  | CataPro (*k*_cat_/*K*_m_) | 0.16 | 0.502 | 0.723 | 0.896 | 1.242 | 0.735 |
| *T*_opt_ | TOMER | 0.63 | 0.414 | **0.704** | **10.50** | **14.19** | 0.637 |
|  | Seq2Topt (*T*_opt_) | 0.57 | **0.431** | 0.670 | 10.63 | 14.35 | **0.670** |
| *T*_m_ | Seq2Topt (*T*_m_) | 0.64 | **0.716** | **0.849** | **4.94** | **6.74** | **0.788** |
